# Multimodal Therapy with Consolidating Haploidentical Stem Cell Transplantation and Dinutuximab Beta for Patients with High-Risk Neuroblastoma and Central Nervous System Relapse

**DOI:** 10.3390/jcm12196196

**Published:** 2023-09-25

**Authors:** Tim Flaadt, Martin Ebinger, Malin Schreiber, Ruth L. Ladenstein, Thorsten Simon, Holger N. Lode, Barbara Hero, Martin U. Schuhmann, Jürgen Schäfer, Frank Paulsen, Beate Timmermann, Angelika Eggert, Peter Lang

**Affiliations:** 1 Department of Hematology and Oncology, University Children’s Hospital, Eberhard Karls University Tuebingen, 72076 Tuebingen, Germany; martin.ebinger@med.uni-tuebingen.de (M.E.); malin.schreiber@med.uni-tuebingen.de (M.S.); peter.lang@med.uni-tuebingen.de (P.L.); 2 Department of Pediatrics, St Anna Children’s Hospital, Medical University, 1090 Vienna, Austria; ruth.ladenstein@ccri.at; 3 Studies and Statistics of Integrated Research and Projects, Children’s Cancer Research Institute, 1090 Vienna, Austria; 4 Department of Pediatric Oncology and Hematology, University Hospital, University of Cologne, 50937 Köln, Germany; thorsten.simon@uk-koeln.de (T.S.); barbara.hero@uk-koeln.de (B.H.); 5 Department of Pediatric Hematology and Oncology, University Medicine Greifswald, 17489 Greifswald, Germany; holger.lode@med.uni-greifswald.de; 6 Section of Pediatric Neurosurgery, Department of Neurosurgery, University Hospital of Tuebingen, 72076 Tuebingen, Germany; martin.schuhmann@med.uni-tuebingen.de; 7 Department for Diagnostic and Interventional Radiology, University Hospital, Eberhard Karls University Tuebingen, 72076 Tuebingen, Germany; juergen.schaefer@med.uni-tuebingen.de; 8 Department of Radiation Oncology, University Hospital Tuebingen, Eberhard Karls University Tuebingen, 72076 Tuebingen, Germany; frank.paulsen@med.uni-tuebingen.de; 9 Department of Particle Therapy, University Hospital Essen, West German Proton Therapy Centre Essen (WPE), West German Cancer Center (WTZ), German Cancer Consortium (DKTK), 45147 Essen, Germany; beate.timmermann@uk-essen.de; 10 Department of Pediatric Oncology/Hematology, Charité-Universitaetsmedizin Berlin, 13353 Berlin, Germany; angelika.eggert@charite.de

**Keywords:** dinutuximab beta, neuroblastoma, recurrence, central nervous system

## Abstract

Despite highly intensive multimodality treatment regimens, the prognosis of patients with high-risk neuroblastoma (HRNB) and central nervous system (CNS) relapse remains poor. We retrospectively reviewed data from 13 patients with HRNB and CNS relapse who received multimodal therapy with consolidating haploidentical stem cell transplantation (haplo-SCT) followed by dinutuximab beta ± subcutaneous interleukin-2 (scIL-2). Following individual relapse treatment, patients aged 1−21 years underwent haplo-SCT with T/B-cell-depleted grafts followed by dinutuximab beta 20 mg/m^2^/day × 5 days for 5–6 cycles. If a response was demonstrated after cycle 5 or 6, patients received up to nine treatment cycles. After haplo-SCT, eight patients had a complete response, four had a partial response, and one had a stable disease. All 13 patients received ≥3 cycles of immunotherapy. At the end of the follow-up, 9/13 patients (66.7%) demonstrated complete response. As of July 2023, all nine patients remain disease-free, with a median follow-up time of 5.1 years since relapse. Estimated 5-year event-free and overall survival rates were 55.5% and 65.27%, respectively. Dinutuximab beta ± scIL-2 following haplo-SCT is a promising treatment option with a generally well-tolerated safety profile for patients with HRNB and CNS relapse.

## 1. Introduction

Neuroblastoma, the most common extracranial solid tumor in childhood, accounts for 15% of all cancer-related deaths in people <14 years of age [1]. Approximately 50% of patients with neuroblastoma exhibit a high-risk phenotype (HRNB), characterized by metastases in patients >18 months of age or the presence of an amplified MYCN gene in patients of any age [2,3]. The 5-year overall survival (OS) rate among these patients is ~60% [4,5], but approximately half of these patients relapse [6]. 

Metastatic recurrences represent a therapeutic challenge in HRNB, particularly in patients with central nervous system (CNS) disease [7]. CNS relapses have been reported between 6 and 11% of patients with neuroblastoma [8,9,10]. The median OS of patients with CNS disease is <6 months, and only 10% of patients survive 36 months, an outcome that did not improve appreciably between 1977 and 2017 [11]. The risk of CNS metastases is linked to both baseline patient characteristics (female sex) and disease characteristics (MYCN amplification, liver metastases, >1 metastatic site), and is unaffected by prior high-dose chemotherapy or immunotherapy [10]. There is currently no established therapy for patients with HRNB and CNS relapse. Various multimodal therapy approaches have been evaluated. One of these approaches is compartmental radioimmunotherapy (cRIT) incorporating intrathecal ^131^I-monoclonal antibodies, which has been investigated in a study conducted by the Memorial Sloan Kettering Cancer Center (MSKCC; NCT00445965/NCT00089245) [7,12]. Local therapy alone—such as surgery, radiation, or local radioimmunotherapy—is unlikely to be sufficient and will need to be followed by systemic therapy. Immunotherapeutic approaches have been implemented in the frontline and relapsed settings to improve outcomes in patients with HRNB [13], with monoclonal antibody therapy directed against the disialoganglioside GD2 being the most established option. The human–mouse–chimeric monoclonal anti-GD2 antibody dinutuximab beta is approved in Europe as post-consolidation therapy for patients with HRNB [14]. Treatment with anti-GD2 antibodies is also well-established in the treatment of relapsed disease [5,14], but its role in the treatment of CNS relapses is unclear [5,15]. Due to its structure and size, dinutuximab beta is unlikely to penetrate a healthy blood–brain barrier [10,16]. However, evidence suggests that in the case of cerebral metastasized disease, the blood–brain barrier may be disturbed due to either therapy or the tumor itself, which can lead to relevant concentrations of therapeutic antibodies in the area of the metastasis [17,18]. Nevertheless, this has not been demonstrated for dinutuximab beta or other systemically given anti-GD2 antibodies. 

We previously reported the results of our feasibility study evaluating the use of immunotherapy with the anti-GD2 antibody dinutuximab beta and subcutaneous interleukin 2 (scIL-2) after haploidentical stem cell transplantation (haplo-SCT) in patients with relapsed metastatic neuroblastoma [19]. This consolidating therapy regimen was administered in 13 patients with HRNB and CNS relapses, including 10 patients who participated in the feasibility study. Here, we report the details of the therapy regimen and the outcomes of these 13 patients. 

## 2. Materials and Methods

### 2.1. Study Design and Patients

We retrospectively reviewed data from 13 patients with HRNB and CNS relapse who received consolidating dinutuximab beta ± scIL-2 following haplo-SCT between September 2010 and October 2021. Ten of these patients were part of the prospective multicenter, multinational, open-label, Phase I/II study (NCT02258815) that aimed to evaluate the safety, feasibility, and survival outcomes of treatment with dinutuximab beta plus scIL-2 after haplo-SCT in intensively pretreated patients with relapsed neuroblastoma [19]. 

In the prospective feasibility study, patients aged 1–21 years with either International Neuroblastoma Staging System (INSS) stage 4 neuroblastoma or INSS stage 2–3 neuroblastoma with MYCN amplification who had relapsed following standard therapy (autologous or allogeneic SCT), or patients with primary refractory stage 4 neuroblastoma, received dinutuximab beta immunotherapy plus scIL-2 following haplo-SCT. A total of 70 patients were enrolled between 11 November 2010, and 26 November 2017, 10 (14.3%) of whom experienced CNS relapse. 

CNS relapse was defined as the appearance of a leptomeningeal or parenchymal lesion confirmed by magnetic resonance imaging (MRI). Metastases originating from the bone of the skull were only included if clear leptomeningeal spread was confirmed.

After the feasibility study was closed, three additional patients with HRNB and CNS relapse also received dinutuximab beta immunotherapy following haplo-SCT in a prospective registry and were included in this analysis.

### 2.2. Treatment

Prior to undergoing haplo-SCT, patients had received various CNS-directed chemotherapies and, if possible, surgery, local radiotherapy, and/or ^131^I meta iodobenzylguanidine (mlBG) therapy. Haplo-SCT was carried out as previously described [19]. All patients received a myeloablative conditioning regimen with fludarabine (40 mg/m^2^, days −8 to −5), thiotepa (2 × 5 mg/kg, day −4), melphalan (70 mg/m^2^, days −3 and −2), and anti-thymocyte globulin (ATG Fresenius Grafalon, Graefelfing, Germany) 30 mg/kg starting on days −12 to −9. OKT3 was given as graft rejection prophylaxis in some patients; however, it was not available after 2013 and was replaced with ATG. All patients received granulocyte colony-stimulating factor (G-CSF) mobilized peripheral blood stem cells from full haplotype identical donors. The stem cell apheresis product was depleted of CD3+ and CD19+ and later TCRαβ+ and CD19+ cells using automated cell sorting, as described previously [20,21,22]. Mycophenolate mofetil (1200 mg/m^2^/day) was administered as post-transplant prophylaxis against graft-versus-host disease (GvHD) until day +30 if residual graft T cells were >2.5 × 104/kg.

Dinutuximab beta was initiated 60–180 days post-transplant if the patient had no signs of GvHD and did not require immunosuppressant medication. The initial regimen comprised 6 consecutive 4-week cycles of 20 mg/m^2^ dinutuximab beta administered intravenously over 8 h per day on the first 5 days of each cycle. Low-dose scIL-2 (1 × 106 IU/m^2^/d) was administered on days 6, 8, and 10 of cycles 4–6 in patients with no signs of severe acute GvHD (grade 3/4) or extensive chronic GvHD. Patients with a response (complete response (CR), partial response (PR), or stable disease (SD)) after 6 cycles of dinutuximab beta were eligible to receive up to 3 additional cycles (maximum 9 cycles in total). 

The three patients who were not part of the feasibility study also underwent haplo-SCT using the same protocol, but received only 5 cycles of dinutuximab beta, in line with the Summary of Product Characteristics [14]. ScIL-2 was omitted from the treatment regimen in these three patients based on recent data from the International Society of Pediatric Oncology, European Neuroblastoma (SIOPEN) trials demonstrating that IL-2 is associated with increased toxicity [5].

### 2.3. Study Assessments

Tumor response was assessed prior to and after haplo-SCT, after cycles 3, 5, or 6, and 9 (if applicable) of dinutuximab beta; then, after 1 year, and then annually. 

Evaluations included mIBG-scintigraphy (SIOPEN- mIBG score), bone marrow (BM) aspirates, and whole-body MRI or MRI/computed tomography (CT) scans of tumor sites, according to the Response Evaluation Criteria in Solid Tumors (RECIST). BM samples were analyzed using microscopy and minimal disease (MD) evaluation with automatic immunofluorescence detection of GD2/CD56-positive neuroblastoma cells, according to Mehes et al. [23] and later published international guidance [24].

### 2.4. Statistical Analysis

Patient characteristics and side effects were analyzed descriptively. Event-free survival (EFS; events defined as relapse, progressive disease (PD), death, or second malignancy) and OS were estimated using the Kaplan–Meier method, starting from the beginning of treatment, i.e., the first day of the first dinutuximab beta cycle after haplo-SCT. Statistical analyses were performed using SPSS^®^ Statistics version 27 for Mac (IBM, Armonk, NY, USA).

## 3. Results

### 3.1. Patient Characteristics

Key patient details and characteristics of the primary tumors in all patients are shown in Table 1, along with the treatment they received for their primary tumors. The median age at diagnosis was 3 years and 5 months (range of 5 months to 7 years and 2 months), and the majority of patients were male (9/13; 69%). Most (11/13; 85%) patients had stage 4 neuroblastoma at diagnosis; one patient had stage 3 neuroblastoma with MYCN amplification, and one was initially classified as stage 4S neuroblastoma but was then reclassified as stage 4 after a subsequent relapse. Four patients (31%) were previously treated with dinutuximab beta post-consolidation therapy. 

CNS relapses occurred after a median of 19 months (range 12–82 months) after initial diagnosis. Five patients had isolated CNS disease and eight patients had combined relapses (CNS and systemic) (Table 2). Five patients with CNS disease had affection of the spine, and seven patients had leptomeningeal involvement.

### 3.2. Treatment Outcomes

**Figure 1:**
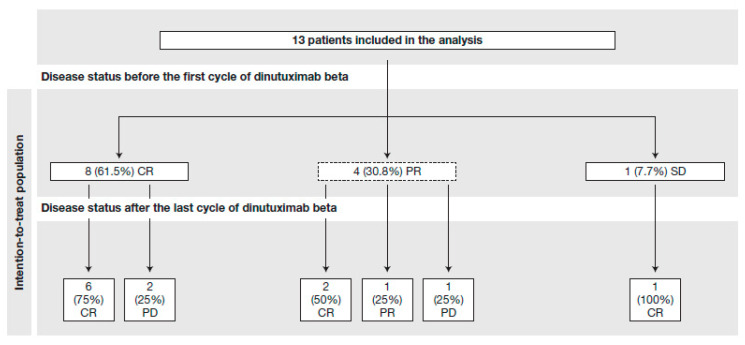
Disease status before and after dinutuximab beta treatment. CR, complete response; PD, progressive disease; PR, partial response; SD, stable disease.

Following the detection of CNS metastases, all 13 patients underwent individualized multimodal treatment for their relapse(s). Four patients had complete and five incomplete resections of the brain lesions, nine patients received mIBG therapy, and eleven patients received radiotherapy (craniospinal irradiation with local boost, n = 5; whole brain irradiation, n = 2; focal irradiation of the metastasis, n = 4). Remission status after haplo-SCT was CR in eight patients (61.5%), PR in four patients (30.8%), and SD in one patient (7.7%) (Table 2 and Figure 1). 

All thirteen patients went on to receive a median of six cycles of dinutuximab beta ± scIL-2 (range 3–9 cycles). After the completion of immunotherapy, the majority of patients (9/13; 61.5%) demonstrated CR, one of whom experienced a CNS relapse within 6 weeks of completing therapy but achieved CR following additional neurosurgery, craniospinal radiotherapy plus local boost with a total dosage of 40 Gy and concomitant chemotherapy with carboplatinum. As of July 2023, all nine patients who were in CR after immunotherapy are still free of disease, with a median follow-up time of 5.1 years (range 0.9–11.5) since CNS relapse. In four out of five patients (80%) with residual disease after haplo-SCT, the tumor load was reduced or maintained with dinutuximab beta treatment, with three patients (60%) achieving CR (Figure 1). In one of these patients, a clear response to the intracerebral tumor manifestation was observed during antibody treatment; the patient had received 131I mIBG therapy and local radiotherapy before haplo-SCT. 

**Figure 2:**
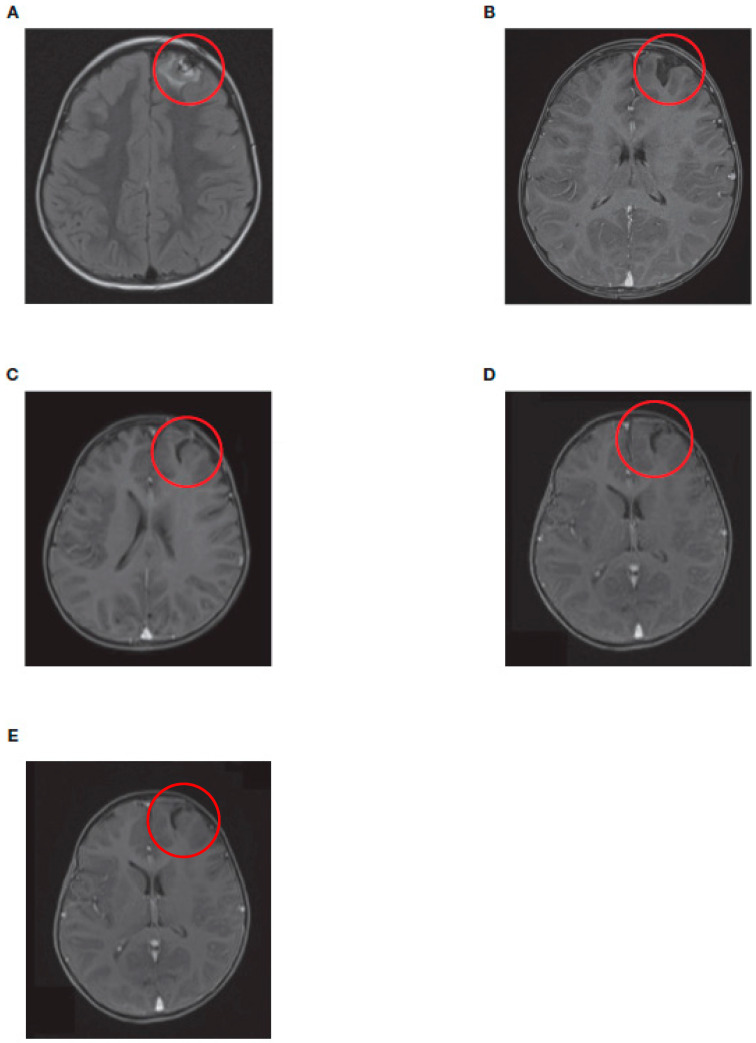
Axial view of brain MRI scans from a patient with neuroblastoma (patient 11) showing intracranial left-frontal metastases at (**A**) initial relapse, (**B**) after reinduction chemotherapy and radiotherapy, (**C**) after haplo-SCT, (**D**) after dinutuximab beta treatment completion, and (**E**) 18 months after haplo-SCT. Red circles show the site of metastases. Haplo-SCT, haploidentical stem cell transplantation; MRI, magnetic resonance imaging.

Brain MRI scans from one of the patients (patient 11) illustrate the ongoing resolution of a singular intracranial left-frontal relapse throughout the course of treatment (Figure 2). Four months after autologous SCT, the patient developed an asymptomatic relapse in the brain parenchyma and the lumbar spine, which were initially treated with relapse chemotherapy with irinotecan/temozolomide (I/T). After four cycles, the CNS metastasis and the metastases in the lumbar region were irradiated with 21 Gy and 36 Gy, respectively. Surgery was not performed after individual risk assessment and parental wish. After the completion of radiotherapy, two further cycles of I/T were given, resulting in CR. The next patient received haplo-SCT followed by five cycles of dinutuximab beta. Both treatments were well-tolerated. The patient has been in sustained remission since then and has not experienced any late neurological effects. The patient is slowly recovering from severe cachexia, which started during primary therapy.

**Figure 3:**
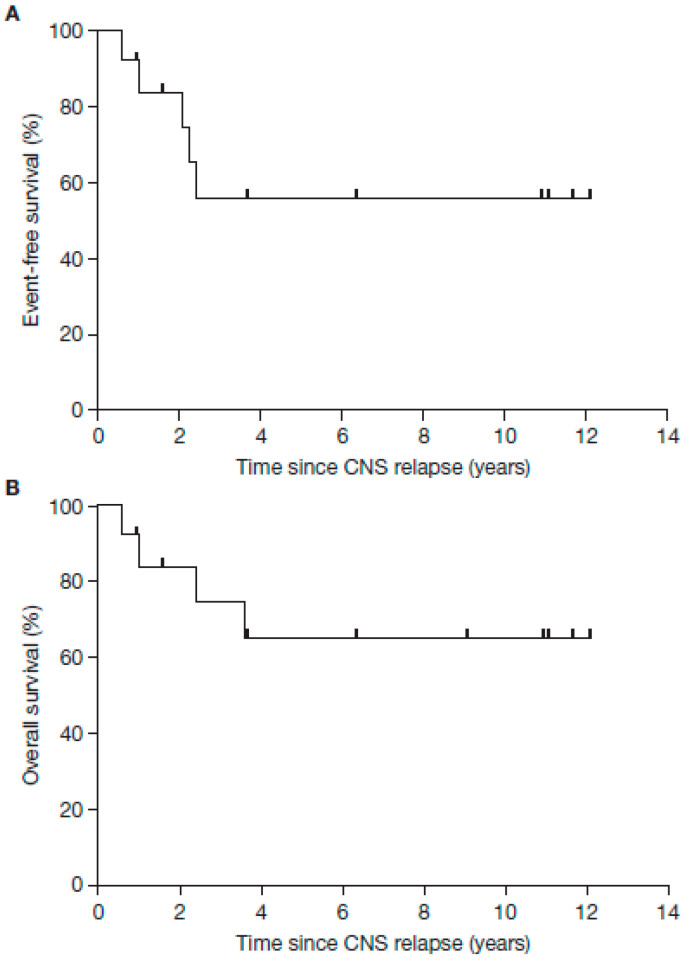
Kaplan–Meier curves demonstrating EFS (**A**) and OS (**B**) in patients with neuroblastoma and CNS relapse treated with haplo-SCT and dinutuximab beta therapy as part of multimodal therapy. Five-year EFS for the whole cohort (years since CNS relapse): 55.9% (95% CI 78.9–23.9); 5-year OS for the whole cohort (years since CNS relapse): 65.3% (95% CI 85.5–31.5). CNS, central nervous system; CI, confidence interval; EFS, event-free survival; haplo-SCT, haplo-identical stem cell transplantation; OS, overall survival.

EFS and OS probabilities for this cohort are shown in Figure 3A,B. The 5-year EFS and 5-year OS rates from the start of relapse treatment were 55.9% (95% confidence interval (CI) 78.9−23.9) and 65.3% (95% CI 85.5−31.5), respectively.

### 3.3. Safety

Treatment-related adverse events (AEs) are summarized in Table 3. Hematologic grade 3/4 AEs occurred in eight patients (10.5%) or twenty-four of seventy-six cycles of dinutuximab beta (31.6%), with hemolytic anemia reported in one patient. The most common non-hematologic grade 3/4 AEs were those affecting the patient’s general condition, allergies, central neurotoxicity, and diarrhea.

One patient discontinued dinutuximab beta treatment due to a serious AE after cycle 7 (chronic hemolysis; patient 2). The patient died as a result of Escherichia coli sepsis but had no tumor progression. One patient died of a secondary malignancy (B-acute lymphoblastic leukemia) 1 year after the end of the study. Both deaths were considered to be associated with the intense pre-therapy and/or haplo-SCT. Two additional patients died due to progressive disease after completion of the study treatment. No patient experienced GvHD during dinutuximab beta treatment.

### 3.4. Therapy Recommendations

Table 4 presents a possible sequence and recommendations for the individual therapy elements. Nevertheless, it is difficult to issue generally applicable therapy recommendations in such a highly complex situation. Therefore, discussing the individual patient in an interdisciplinary conference with national and, if necessary, international experts is recommended.

## 4. Discussion

Therapeutic options for children with HRNB and CNS relapse are limited, and the prognosis is poor [10], indicating the need for new treatment approaches. Multimodal treatment strategies have proven essential in the treatment of patients with HRNB and CNS relapse. They typically consist of locally acting therapy, such as surgery and radiation, followed by reinduction chemotherapy regimens that are able to target the CNS directly, including irinotecan and temozolomide [7]. Radiotherapy is considered to be necessary, with current recommendations favoring craniospinal irradiation over local radiotherapy [26]. Surgery is also recommended in most cases, partly to evaluate the possibility of targeted therapy. Our findings suggest that the addition of subsequent consolidating haplo-SCT followed by immunotherapy with dinutuximab beta, with or without scIL-2, may have additional benefits in this patient group. Retrospective analysis of data from 13 children with HRNB and CNS metastases at relapse who were treated with this regimen demonstrated a positive impact on tumor response rates and survival outcomes. 

The incidence of CNS relapse in HRNB is relatively low [16]. In the prospective feasibility study described here [19], 14.3% of the HRNB patients who relapsed had CNS involvement, which is higher than the ~6% previously reported [10]. The median age at diagnosis of patients with CNS relapse was considerably lower than the whole cohort from the feasibility study (3 years 4 months versus 6 years 6 months), which included those with and without CNS involvement [19]. A recent study suggested that certain patient and disease characteristics such as female sex, MYCN amplification, hepatic and >1 metastatic system/compartment involvement, are associated with increased risk of CNS relapse [10]; however, in contrast to previous reports [16], a prior high-dose chemotherapy regimen or the use of immunotherapy did not impact on the occurrence of CNS relapse [10].

So far, there is no established therapy to prevent CNS relapses, and the need for such prophylactic treatment is controversial [10]. A topotecan-based myeloablative regimen was unable to prevent CNS relapse [27]. In addition, not much progress has been made in the treatment of CNS relapses. Despite multimodal regimens including surgery, radiotherapy, and chemotherapy showing effectiveness in controlling tumor progression from localized CNS metastases, remissions have been seen to be temporary, with the median survival from the time of CNS recurrence ranging from 1 to 5 months [28,29,30]. The MSKCC investigated a multimodal treatment approach using cRIT with intrathecal 131I-monocolonal antibodies targeting GD2 or B7H3 following surgery, craniospinal irradiation, and chemotherapy, which demonstrated favorable survival in patients with relapsed CNS neuroblastoma [7,12]. However, the impact of cRIT in the multimodality treatment has so far not been investigated in a comparative prospective clinical trial. 

Our treatment approach resulted in CR for the majority of patients (9/13) and was generally well-tolerated, with manageable side effects. The side effects were comparable to those of the entire cohort from our Phase I/II feasibility study and differed slightly from previous reports [19,31]. This may be due to the relatively small number of patients included in our study and the absence of specific side effects. In particular, the rate of CNS toxicity was relatively low in the present cohort. The occurrence of hemolytic anemia, a side effect that has already been described in the context of anti-GD2 therapy after haplo-SCT [19], was also low in this patient group. While four patients died as a result of either disease progression (n = 2, one patient with CNS progression, one patient with metastatic disease outside the CNS) or serious AEs that were unrelated to dinutuximab beta treatment (n = 2), the remaining patients are still free of disease as of July 2023, with a median follow-up time of 5.1 years (range 0.9–11.5) since CNS relapse and estimated EFS and OS rates of 55.9% and 65.3%, respectively. 

Although we were able to demonstrate a positive effect with our dinutuximab beta-containing treatment regimen, it is unclear whether the antibody is able to penetrate the blood–brain barrier and exert its action directly in the CNS. It remains uncertain if the response on the intracerebral metastasis during dinutuximab beta treatment observed in one patient was due to delayed effects of chemotherapy and mIBG treatment, or if the antibody itself caused this effect. While under normal circumstances, dinutuximab beta does not cross the blood–brain barrier, and it is possible that either the tumor itself or prior therapy, such as radiation and/or surgery, may cause disruption to the blood–brain barrier, potentially enabling penetration of the monoclonal antibody. Regardless of any direct effects on the CNS, consolidating immunotherapy with dinutuximab beta following haplo-SCT may be beneficial for the treatment of other tumor manifestations and minimal residual disease. As shown in our feasibility study, such a treatment regimen is associated with encouraging EFS and OS outcomes in patients with relapsed HRNB, including those with CNS relapse [18]. Based on our data, we cannot conclude whether surgery is absolutely necessary, yet in most cases, it is recommended to also evaluate the possibility of a targeted therapy. Nevertheless, in our study, we observed cases in which surgery with tumor spillage resulted in further CNS metastasis. It is, therefore, advisable to administer chemotherapy and/or radiotherapy after surgery.

Limitations of our study include the small number of patients involved and the retrospective nature of the analysis, which is due to the rarity of the disease as well as the experimental nature of this treatment approach. The effectiveness and safety of dinutuximab beta have recently been demonstrated in a much larger cohort of patients with frontline or relapsed/refractory HRNB in real-world clinical practice [13]. Additionally, our cohort includes patients with relapsed HRNB who did not progress rapidly while receiving individual relapse treatment, and the side effects of relapse treatment prior haplo-SCT were not systematically assessed. Despite these limitations, multimodal therapy with consolidating dinutuximab beta following haplo-SCT appears to be a promising treatment option for patients with relapsed HRNB, including those with CNS relapse. This treatment strategy was associated with a complete remission of CNS metastases in the majority of patients, with encouraging effects on EFS and OS, and was generally well-tolerated. Further studies are needed to evaluate this approach in a wider clinical setting.

## Figures and Tables

**Table 1:** Key patient and disease characteristics and treatment received for the primary tumor in patients with HRNB who went on to have a CNS relapse.

Pt	Sex	Age at Diagnosis	INSS Stage	Details of Primary Tumor (And Metastases)	MYCN Status	mIBG Status	Initial Treatment for Primary Tumor
1	M	3 yrs 4 m	4	Abdominal and thoracic Bone metastases (right ileac); BM involvement	Amplified	+	Chemotherapy (ANBL0532) Resection Tandem high-dose therapy and ASCT Local radiotherapy Immunotherapy: six cycles of DB and isotretinoin
2	M	3 yrs 4 m	4	Paravertebral border cord	Not amplified	+	Chemotherapy (NB2004-HR)
3	M	2 yrs 3 m	3	Left adrenal gland	Amplified	+	Chemotherapy (NB2004-HR)
4	F	3 yrs 7 m	4	Left pre- and paravertebral Metastases in cranial bones, scapula, humerus, ulna, ribs, spine, pancreas, cervical, and supraclavicular lymph nodes; BM involvement	Not amplified	+	Chemotherapy (NB2004-HR)
5	M	2 yrs 3 m	4	Abdominal Bone metastases	Not amplified	+	Chemotherapy (NB2004-HR)
6	M	1 yr 5 m	4	Left adrenal gland Cervical, retrobulbar metastases; liver involvement; BM involvement	Amplified	+	Chemotherapy (NB2004-HR)
7	M	7 yrs 2 m	4	Left retroperitoneal Metastases in the spine and lymph node involvement	Unknown	+	Chemotherapy (NB97-MR)
8 ^a^	M	5 m	4S	Left adrenal gland Osseous, pulmonary, and orbital metastases with infiltration of skull base and epidural infiltration; BM involvement	Not amplified	+	Watch and wait, then chemotherapy (NB2004-HR)
9	M	3 yrs 9 m	4	Left adrenal gland Orbital to intracranial fronto-temporal (left) metastases	Amplified	+	Chemotherapy: rapid COJEC Resection Tandem high-dose therapy and ASCT Radiotherapy (primary tumor and orbital left) Immunotherapy: thee cycles of DB + IL-2 s.c. + isotretinoin
10	F	4 yrs 11 m	4	Right adrenal gland Diffuse osseous metastases; BM involvement	Not amplified	+	Unknown ^b^
11	M	4 yrs	4	Retroperitoneal Disseminated osseous metastases; BM infiltration	Not amplified	+	Chemotherapy (NB2004-HR)
12	F	3 yrs 1 m	4	Retroperitoneal Disseminated osseous metastases; BM infiltration	Not amplified	+	Resection ASCT Radiotherapy Immunotherapy: six cycles of isotretinoin and five cycles of DB beta
13	F	3 yrs 7 m	4	Left adrenal gland Lymph nodes and cervical and supraclavicular bones	Amplified	+	Chemotherapy (NB2004-HR) ASCT Immunotherapy: five cycles of DB

^a^ Patient 8 was reclassified as stage 4 after a subsequent relapse; ^b^ primary tumor was not treated at our center. yr(s), years; m, months ANBL0532, two cycles topotecan/cyclophosphamide, with four alternating cycles of cisplatin/etoposide, and cyclophosphamide/doxorubicin/vincristine; ASCT, autologous stem cell transplantation; BM, bone marrow; CNS, central nervous system; COJEC, cisplatin, carboplatin, cyclophosphamide, vincristine, etoposide; DB, dinutuximab beta; F, female; HR, high risk; HRNB, high-risk neuroblastoma; IL-2, interleukin-2; INSS, International Neuroblastoma Staging System; M, male; mIBG; iodine-131-metaiodobenzylguanidine uptake; MR, medium risk; NB2004-HR, six cycles of alternating N5 and N6 cycles; N5 (cisplatin, etoposide, vindesine) and N6 (vincristine, dacarbacine, ifosfamide, doxorubicin), no patient was randomized to receive two cycles of N8 (topotecan, cyclophosphamide, etoposide); NB97-MR, six cycles of alternating N5 (cisplatin, etoposide, vindesine) and N6 (vincristine, dacarbacine, ifosfamide, doxorubicin) for NB2004-HR; s.c., subcutaneous.

**Table 2:** Key patient and disease characteristics and treatment received for relapse in patients with HRNB and CNS relapse.

Pt	TTFR, m	Details of Relapse	Treatment (Prior to Haplo-SCT and DB)	Status Before Haplo-SCT	Donor	Status after Haplo-SCT	DB Cycles (n) ^a^	Status of Disease after DB Cycle:			Last Known Status
								3	5/6	9	
1	17	Distant metastases (CNS only) Temporopolar dural metastasis (right cranial fossa) with local hemorrhage	Complete resection of dural metastasis CSI 21.4 Gy mIBG (7.4 GBq)	PR	Mother	CR	6	CR	CR	–	CR; 15 yrs 11 m of age (19 August 2021)
2	14	Distant recurrence (bone/BM, CNS) Skull fronto central and left occipital CNS in the area of resection margin Systemic (proximal femur right, trochanter major left, ilium right, right humeral head)	Resection of CNS metastases Chemotherapy: 3 × N8, 2 × RIST (I/T) Whole brain RT (CSI up to 21.6 Gy, boost to metastasis to cum 31.2 Gy) Microscopic tumor resection right frontal, Ommaya-reservoir left frontal Chemotherapy (3 × N8) mIBG (6.45 GBq)	MR	Father	PR	7	PR	PR	–	Seven cycles of DB were given; stopped due to chronic hemolysis The patient died Nine months after seventh cycle of DB due to sepsis (*E. coli*); no tumor progression (7 yrs 5 m of age)
3	19	Distant metastases (CNS only) First relapse: isolated cerebellar metastasis left Second relapse: intraspinal metastasis Th5/6 with cross-sectional symptoms and spinal S1; cerebellar metastasis right; blastomatous meningiosis	First relapse: Complete resection Chemotherapy 2 × N8, 2 × I/T, high-dose busulfan/melphalan ASCT Second relapse: Laminotomy Th5/6, partial removal of tumor (decompression) CSI (30.6 Gy) 4 × intrathecal topotecan mIBG (6.54 GBq)	PR	Father	PR	9	CR	CR	CR	CR; 15 yrs 8 m of age (24 September 2021)
4	27	Distant metastases (CNS only) Intracranial right frontal, left temporal, left frontobasal, involvement of the brain parenchyma, large liquid/hemorrhagic formations,	Partial tumor resection Whole brain RT (up to 30 Gy, boost to residue to cum. 36 Gy) with simultaneous chemotherapy (melphalan) mIBG (8.3 GBq)	CR	Mother	CR	7	CR	CR	–	DB + IL-2 was stopped after seven cycles at the parent’s request CR; 16 yrs 10 m of age (15 September 2021)
5	25	Distant recurrence only (bone, CNS) Left temporoparietal cranial metastasis, progressive metastases in both maxillary sinuses Bone metastases: right femur, right humerus	Chemotherapy (RIST × 2) Resection of the left adrenal gland (histology: mature ganglioneuroma) mIBG (8.77 GBq)	CR	Mother	CR	9	CR	CR	CR	The patient relapsed (cerebral progression) within 6 weeks of completing DB After surgery and radiotherapy, the patient achieved CR CR; 12 yrs 7 m of age (14 September 2021)
6	16	Distant recurrence only (bone, CNS) Right temporal	Partial resection (hemorrhage, symptomatic upper entrapment) Chemotherapy (RIST × 1) mIBG (dosage unknown) Local RT	PR	Mother	CR	6	CR	CR	–	CR; 9 yrs 1 m of age (1 July 2021)
7	31	Local and distant recurrences (bone, BM, CNS) First relapse: parameningeal skull base, liver, BM Second relapse: skull base left, left humerus, right femur, right tibia, pelvis Third relapse: left temporopolar, os ilium Fourth relapse: diffuse osteomedullary (filiae scapula right, Th8, distal femur left, proximal tibia right, Th9), skull base with CNS involvement	Relapses 1–3: Chemotherapy mIBG (300 mCi) Radiotherapy (first 36 Gy skull base, second 39.6 Gy) ASCT Fourth relapse: Chemotherapy (RIST × 7) Local RT (38.4 Gy) mIBG (10.2 GBq)	PR	Mother	SD	9	PR	PR	CR	CR; 22 yrs of age (16 July 2021)
8	82	Local and distant recurrences (bone, BM, CNS) First relapse: osseous, pulmonary, orbital metastases, infiltration of the skull base, epidural spread, BM infiltration Second relapse: disseminated; progression of existing lesions, new metastases on the lateral right orbital wall, right mediastinal, abdominal retroperitoneal	First relapse: Chemotherapy (NB2004-HR) Resection mIBG (8 GBq) High-dose chemotherapy ASCT Isotretinoin, 3 × DB (LTI) (discontinued due to relapse/progression) Second relapse: Chemotherapy (RIST × 12) Local RT (40 Gy)	PR	Mother	PR	6 ^a^	PR	PD	–	The patient died of tumor progression (5 months after receivingthe sixth cycle of DB)
9	12	First relapse Distant recurrence only (bone, CNS) Multiple leptomeningeal and intraspinal Second relapse Intracranial, multiple intraspinal	First relapse: Relapse noticed after the third DB cycle during treatment of primary tumor Chemotherapy (TVD + thiotepa × 6) CSI (21 Gy) mIBG Second relapse: RT of posterior fossa and spinal axis 22 Gy) Isotretinoin p.o.	PR	Mother	CR	6	CR	PD	–	The patient relapsed shortly after receiving the sixth cycle of DB (2 × intracranial and multiple intraspinal) Received radiotherapy to the cerebellar region and spinal axis and isotretinoin The patient died 6 months later
10	Unknown ^b^	Distant recurrence only (other, CNS) Cerebral relapse, paraventricular right mass of the caudate nucleus	Chemotherapy (I/T × 4) Biopsy CSI (29 Gy)	PR	Mother	CR	3	PD	–	–	The patient discontinued treatment due to progressive disease (brain) noted during restaging after the third cycle DB The patient died of B-ALL (secondary malignancy) 6 months later
11	14	Distant recurrence only (bone, BM, CNS) Intracranial left-frontal Lumbar vertebrae	Chemotherapy (I/T) Local RT (primary tumor 21 Gy, metastases 36 Gy)	CR	Father	CR	5	CR	CR^c^	–	CR; 9 yrs of age (January 2023)
12	19	Local and distant recurrences (bone, BM, lymph nodes, CNS) Paravertebral T6-11, intraspinal T7-T8, with spinal compression, BM, mediastinal lymph nodes, osseous vertebrae, os sacrum, pelvis, femur, scapulae Cranial suprasylvian left New lesions sternum, os sacrum	Incomplete resection Chemotherapy (TVD × 4) Local RT (Th9/Femur li, brain 21 Gy, left-frontal 36 Gy, sternal 30 Gy, sacral 30 Gy) BEACON neuroblastoma trial (7 × BIT; 1 × I/T + DB)	PR	Mother	PR	5	CR	CR ^c^	–	CR; 7 yrs 6 m of age (January 2023)
13	14	CNS only: parenchymatous cerebral relapse (frontal)	Chemotherapy: 6 × I/T Complete resection 1 × I/T RT (CSI 21.6Gy + tumor boost to com. 36 Gy) + Temozolomide 1 × I/T	CR	Father	CR	5	CR	CR		CR 6 yrs 6 m of age (January 2023)

^a^ All patients received dinutuximab beta via short intravenous infusion (20 mg/m^2^ over 8 h/day on the first 5 days of each cycle) for the treatment of CNS relapse, except patient 8, who received a continuous infusion of 20 mg/m^2^/day 24 h/day on the first 5 days of each cycle; ^b^ patient not treated at our center; ^c^ disease status after the fifth and last cycle of dinutuximab beta; ASCT, autologous stem cell transplantation; B-ALL, B-Cell Acute Lymphoblastic Leukemia; BIT, bevacizumab, irinotecan, temozolomide; BM, bone marrow; CNS, central nervous system; CR, complete remission; CSI, craniospinal irradiation; DB, dinutuximab beta; haplo, haploidentical; HRNB, high-risk neuroblastoma; IL-2, interleukin-2; I/T, irinotecan/temozolomide; LTI, long-term infusion; m, months; mIBG; therapeutic iodine-131-metaiodobenzylguanidine; MR, mixed response; N8, topotecan/cyclophosphamide/etoposide; NB2004-HR, two cycles of N8 (topotecan, cyclophosphamide, etoposide) and six cycles of alternating N5 (cisplatin, etoposide, vindesine) and N6 (vincristine, dacarbacine, ifosfamide, doxorubicin); PD, progressive disease; PR, partial remission; RIST, rapamycin, irinotecan, dasatinib, temozolomide; RT, radiotherapy; SD, stable disease; SCT, stem cell transplant; TTFR, time to first remission; TVD, topotecan (T), vincristine (V), doxorubicin (D); yrs, years.

**Table 3:** Adverse events reported during dinutuximab beta therapy (total number of dinutuximab beta cycles n = 76).

**Adverse Events, n (%)**	**Grade 1/2 ^a^**	**Grade 3/4 ^a^**
General condition	8 (10.5)	4 (5.3)
Diarrhea	9 (11.8)	2 (2.6)
Constipation	12 (15.8)	0 (0)
Stomatitis	9 (11.8)	0 (0)
Skin toxicity	8 (10.5)	0 (0)
Allergy	4 (5.3)	4 (5.3)
Nausea/vomitting	6 (7.9)	1 (1.3)
Central neurotoxicity	2 (2.6)	3 (3.9)
Hypotension	4 (5.3)	1 (1.3)
Pulmonary toxicity	3 (3.9)	0 (0)
Veno-occlusive disease	0 (0)	0 (0)
Cardiac function	0 (0)	0 (0)
QT interval	0 (0)	0 (0)
ECHO: LV-SF	0 (0)	0 (0)
Laboratory abnormalities		
SGOT/SGPT elevation	10 (13.2)	2 (2.6)
Hemoglobin level reduced	8 (10.5)	4 (5.3)
White blood count reduced	5 (6.6)	7 (9.2)
Absolute neutrophil count reduced	2 (2.6)	10 (13.2)
Platelets reduced	5 (6.6)	3 (3.9)
Proteinuria	7 (9.2)	0 (0)
Bilirubin elevation	5 (6.6)	1 (1.3)
Creatinine elevation	5 (6.6)	0 (0)
Decreased GFR (mL/min/1.73 m^2^) ^b^	3 (3.9)	0 (0)
Hemolytic anemia	0	1 (1.3)
Hematuria	1 (1.3)	0 (0)

^a^ Grades according to the Common Terminology Criteria for Adverse Events (CTCAE4.0); ^b^ grade 1/2: <75% to 25% lower level of normal; grade 3/4: <25% lower level of normal (grade 3) to chronic dialysis or renal transplant indicated (grade 4). ECHO: LV-SF, echocardiogram: left ventricular systolic function; GFR, glomerular filtration rate; SGOT, serum glutamic-oxaloacetic transaminase; SGPT, serum glutamic pyruvic transaminase.

**Table 4:** Treatment recommendations for patients with neuroblastoma and CNS relapse.

Surgery	If possible, a resection of the CNS metastasis should be performed initially in order to obtain tissue to evaluate suitability for targeted therapy. Resection after neoadjuvant chemotherapy can be considered in asymptomatic cases in order to reduce the risk of tumor spillage. In our experience, improvement without resection is also possible in case of surgically unfavorably located metastases.
Systemic therapy	CNS-directed chemotherapy should be administered. We had a good experience with irinotecan and temozolomide. Concomitant therapy with dinutuximab beta can be considered, especially in patients with combined relapses. However, the risk for serious neurotoxicity is increased at the start of dinutuximab beta treatment and after surgery in patients with CNS metastases.
Targeted therapy	Targeted therapy, such as *ALK* inhibitors, should be given if indicated. We recommend interrupting therapy during haplo-SCT until stable engraftment is achieved. There are currently no data on concomitant *ALK* inhibitor therapy and radiotherapy in patients with neuroblastoma. Based on reports of adult patients with lung cancer, concomitant use can be considered [25].
Compartmental chemotherapy	Intrathecal chemotherapy, such as etoposide or topotecan, can be considered, especially in patients with leptomeningeal involvement or cancerous meningiosis, or if radiotherapy is not possible.
Radiotherapy	Current recommendations favor craniospinal irradiation with a local boost over the whole brain or local irradiation for neuroblastoma with CNS relapse. In the case of craniospinal irradiation with a local boost, we recommend proton therapy. In case of isolated CNS relapses, early radiotherapy should be administered. If tolerable, concomitant therapy with temozolomide can be considered, especially for patients with combined relapses.
Haploidentical stem cell transplantation	Haplo-SCT should follow previously reported guidance, with myeloablative conditioning with fludarabine, thiotepa, melphalan, and ATG. Transplantation should only be performed if remission status ≥PR is achieved. T- and B-cell-depleted grafts should be used.
Post-transplant-immunotherapy	Dinutuximab beta should be initiated 40–180 days post-transplant if the patient has no signs of GvHD and does not require immunosuppressant medication. According to the Summary of Product Characteristics, five cycles of dinutuximab beta should be given. We do not recommend concomitant therapy with cytokines, like IL-2.
Maintenance therapy	Maintenance therapy with targeted therapy, like *ALK* inhibitors, can be considered.

*ALK*, anaplastic lymphoma kinase; ATG, anti-thymocyte globulin; CNS, central nervous system; GvHD, graft-versus-host disease; haplo-SCT; haplo-identical stem cell transplantation; IL-2, interleukin-2; PR, partial response.

## Data Availability

The original contributions presented in the study are included in the article. Further inquiries can be directed to the corresponding author.
